# Unusual Metastatic Pathways: A Case of Colorectal Adenocarcinoma Presenting as a Cervical Neck Mass

**DOI:** 10.7759/cureus.72379

**Published:** 2024-10-25

**Authors:** Taha Al Hassan, Jessica Flores, Adam Bender-Heine, Rachel A Giese

**Affiliations:** 1 Head and Neck Surgery, University of Texas Rio Grande Valley School of Medicine, Edinburg, USA; 2 General Surgery, University of Texas Rio Grande Valley School of Medicine, Edinburg, USA; 3 Otolaryngology-Head and Neck Surgery, University of Texas Health Science Center at San Antonio, San Antonio, USA

**Keywords:** cervical neck masses, colorectal adeno-carcinoma, head and neck oncology, otolaryngology-head and neck surgery, uncommon metastasis

## Abstract

Head and neck metastasis of colorectal adenocarcinoma is exceedingly rare with most cases presenting in the liver, lungs, or peritoneum. This report describes the case of a 53-year-old female patient with a past medical history significant for mucinous adenocarcinoma of the colon treated with a right hemicolectomy. She was thought to be in remission but presented a few years later with a new, isolated left cervical neck mass and symptoms of left eye ptosis and dryness. Diagnostic imaging and biopsy confirmed the neck mass to be a metastatic lesion from her prior mucinous adenocarcinoma, with immunohistochemical findings specific for colorectal origin. The case highlights the diagnostic challenges posed by such unusual metastatic sites and the importance of considering colorectal cancer in patients with a history of the disease who present with atypical symptoms. Early recognition of metastatic patterns, even in rare locations like the head and neck, is crucial for optimizing treatment strategies, which may include surgical resection, systemic chemotherapy, or targeted therapies. This report emphasizes the need for further research into the mechanisms of metastasis and the development of effective treatment protocols for rare metastatic presentations.

## Introduction

Colorectal cancer (CRC) is one of the most common malignancies worldwide, accounting for roughly 8% of all cancer diagnoses and cancer-related deaths in the United States [1]. Overall, five-year relative survival rates are close to 70%; however, approximately one in five patients present with metastatic disease at the time of initial diagnosis, which is correlated to a reduced five-year survival rate of 15.7% [1]. Common metastatic sites include the liver, lungs, and peritoneum [1,2].

The liver is the most common site, observed in over 70% of metastatic CRC cases, given the direct communication between the colon and the liver through the portal venous system [2]. Second to the liver, lung metastases occur in approximately 15-25% of cases, especially when the tumor originates from the rectum because venous drainage bypasses the liver and connects directly to the lungs through the inferior vena cava [2]. Lastly, the peritoneum is another frequent metastatic site, especially in mucinous and signet-ring cell carcinomas, due to their propensity for transcoelomic spread [2]. While adenocarcinoma is the most common subtype of CRC, the presence of rarer subtypes such as mucinous adenocarcinoma and signet-ring cell carcinoma warrants heightened attention, as these exhibit more aggressive behavior, are associated with a higher risk of distant metastasis, and demonstrate comparatively poorer survival outcomes [2,3].

Overall, metastasis of CRC to other areas remains uncommon, likely due to the absence of connecting vascular or lymphatic pathways, with head and neck metastasis accounting for less than 1% historically [4]. Detecting these rare occurrences is essential for effective patient management, as they introduce significant diagnostic and therapeutic challenges. Clinicians should, therefore, maintain a high index of suspicion for potential metastasis as a differential when evaluating head and neck masses in patients with a history of infraclavicular cancers and no evidence of a primary tumor [5].

This case report comments on the rare presentation of an isolated left cervical neck mass, with immunohistochemical staining confirming its colorectal origin, in a patient with a history of mucinous adenocarcinoma of the colon previously treated with hemicolectomy and believed to be in remission. This case underscores the critical importance of maintaining vigilance and considering a comprehensive differential diagnosis in patients with new left-sided neck masses and a history of CRC. Such an approach ensures that appropriate and proactive diagnostic measures are initiated, which can significantly influence prognosis and treatment outcomes [6].

## Case presentation

A 53-year-old female patient with a past medical history significant for anxiety, gastroesophageal reflux disease, uterine fibroids, and mucinous adenocarcinoma of the colon, post hemicolectomy three years ago, presented for evaluation of a painless but noticeable left cervical neck mass, and acute onset of left eye ptosis and dryness.

Past surgical and pathological findings

In reviewing the patient’s previous reports, it was noted that her cancer was determined to be a moderately differentiated adenocarcinoma with mucinous features. The tumor had extended through the muscularis propria into the pericolic adipose tissue (pT3). At the time of her hemicolectomy, the resected surgical margins were negative for invasive carcinoma, intramucosal carcinoma, or high-grade dysplasia. Of the 16 regional lymph nodes examined during her procedure, four were positive for metastatic carcinoma (pN2a). Further exploration during her procedure identified a splenic flexure colon mass, which was confirmed to be carcinoma in situ arising from a tubulovillous adenoma. While no definitive evidence of invasive malignancy was noted throughout the procedure, the biopsies were superficial, leaving the possibility of an invasive malignancy open.

Current clinical presentation

**Figure 1:**
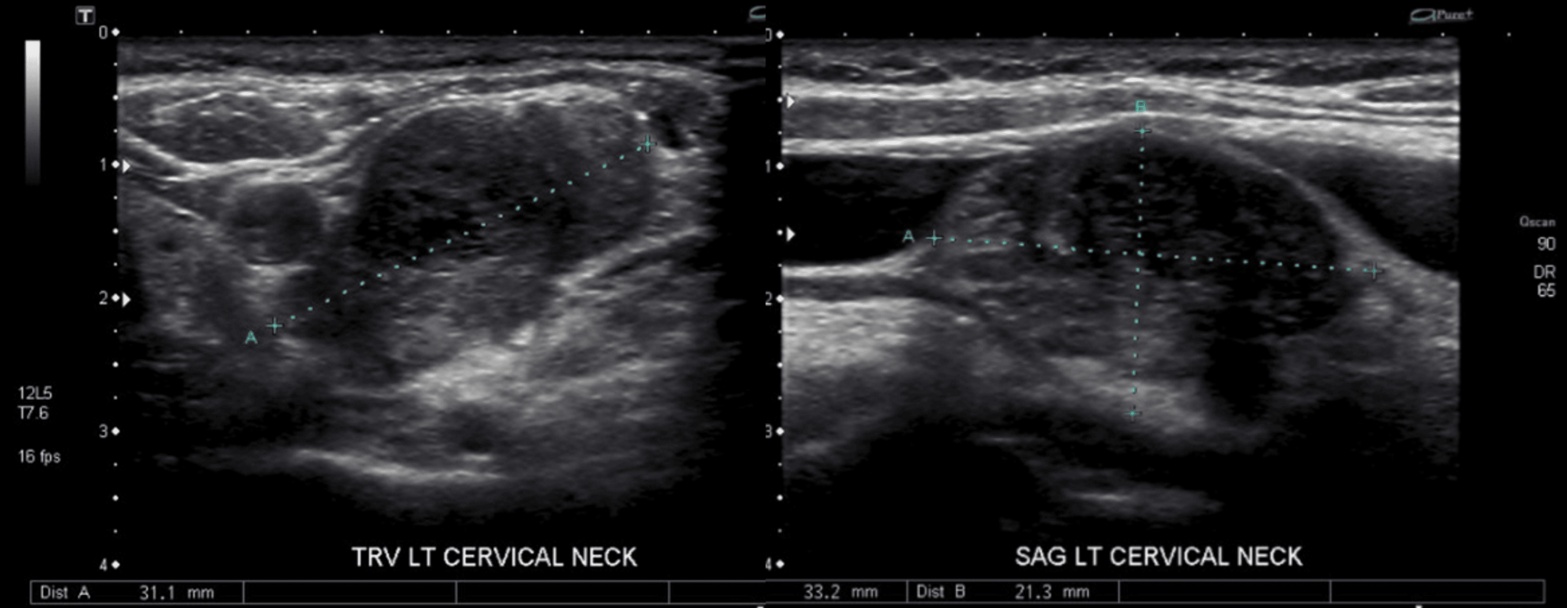
Ultrasound imaging of left cervical neck mass: transverse (left) and sagittal (right) views

On physical examination, a palpable, non-fluctuant left level IV neck mass was identified without accompanying cervical lymphadenopathy. An ultrasound of the neck revealed a right thyroid lobe measuring 4.5 x 1.3 x 1.3 cm, a left thyroid lobe measuring 2.8 x 2.8 x 0.9 cm, and a complex cyst and calcified nodule in the right mid-pole measuring 8 x 5 x 5 mm and 4 x 3 x 4 mm. No discrete thyroid nodule was observed on the left side. The primary finding, however, was a left cervical neck mass measuring 3.3 x 2.1 x 3.1 cm, for which clinical correlation was recommended (Figure 1).

In light of the patient’s history of colorectal adenocarcinoma, no identifiable primary tumor, and the recent development of left eye ptosis and dryness, an ultrasound-guided fine needle aspiration biopsy was performed on the left cervical neck mass for suspicion of malignancy. Pathological analysis confirmed the presence of malignant cells consistent with metastatic colonic adenocarcinoma. Immunohistochemical staining demonstrated partial positivity for CK20 and CDX2, markers typically associated with intestinal origin, while staining was negative for CK7. This immunoprofile strongly supported a colorectal origin [7,8]. Considering the patient’s medical history, the pathological findings, and the specific immunohistochemical results, the neck mass was concluded to be a metastasis from her prior colorectal mucinous adenocarcinoma.

Treatment plan

The proposed treatment plan for the patient included a comprehensive workup, incorporating several imaging studies to assess the extent of metastatic disease. This included a contrast-enhanced CT scan of the chest, abdomen, and pelvis, as well as a PET-CT scan to provide a more detailed evaluation and identify any additional metastatic sites. An MRI of the neck was also recommended to better characterize the cervical mass and assess its relationship with surrounding anatomical structures.

Following the imaging studies, evidence-based systemic chemotherapy options such as FOLFOX (folinic acid, 5-fluorouracil, and oxaliplatin) or FOLFIRI (folinic acid, 5-fluorouracil, and irinotecan) were proposed [9]. These regimens aim to disrupt cancer cell DNA synthesis and division, though, in the process, they also have the potential to damage healthy cells. Depending on the tumor’s genetic profile and microsatellite instability (MSI), targeted therapies such as cetuximab or bevacizumab were to be considered to enhance treatment effectiveness. If the tumor was found to be MSI-high (MSI-H), immunotherapy options like pembrolizumab or nivolumab were also suggested as potential treatments [9]. Lastly, while a surgical consultation was recommended to assess the feasibility of resecting the neck mass, it was clarified that surgery would not be curative, given the metastatic nature of the disease and the likelihood of dissemination to other areas.

Supportive and palliative care measures were integrated into the discussion to potentially manage symptoms and improve quality of life; however, the delayed recognition of the atypical metastatic presentation, combined with the need for a coordinated multidisciplinary approach, complicated the patient’s case and was overwhelming. Unfortunately, despite continuous efforts, she was lost to follow-up and unable to pursue the recommended treatment options due to financial constraints and insurance status. 

## Discussion

Metastasis of CRC to the head and neck region is an exceptionally rare phenomenon, with only a limited number of cases reported in the literature [10-13]. This uncommon presentation introduces significant complexities in both diagnosis and management, as well as emphasizes the importance of maintaining a comprehensive differential diagnosis in patients with past histories of malignancy. The absence of a direct lymphatic or venous connection between the colorectal region and the head and neck complicates the identification of these unusual metastatic sites, which can lead to delayed care or missed diagnoses. Early recognition could significantly influence management strategies, prognosis, and overall outcomes.

**Figure 2:**
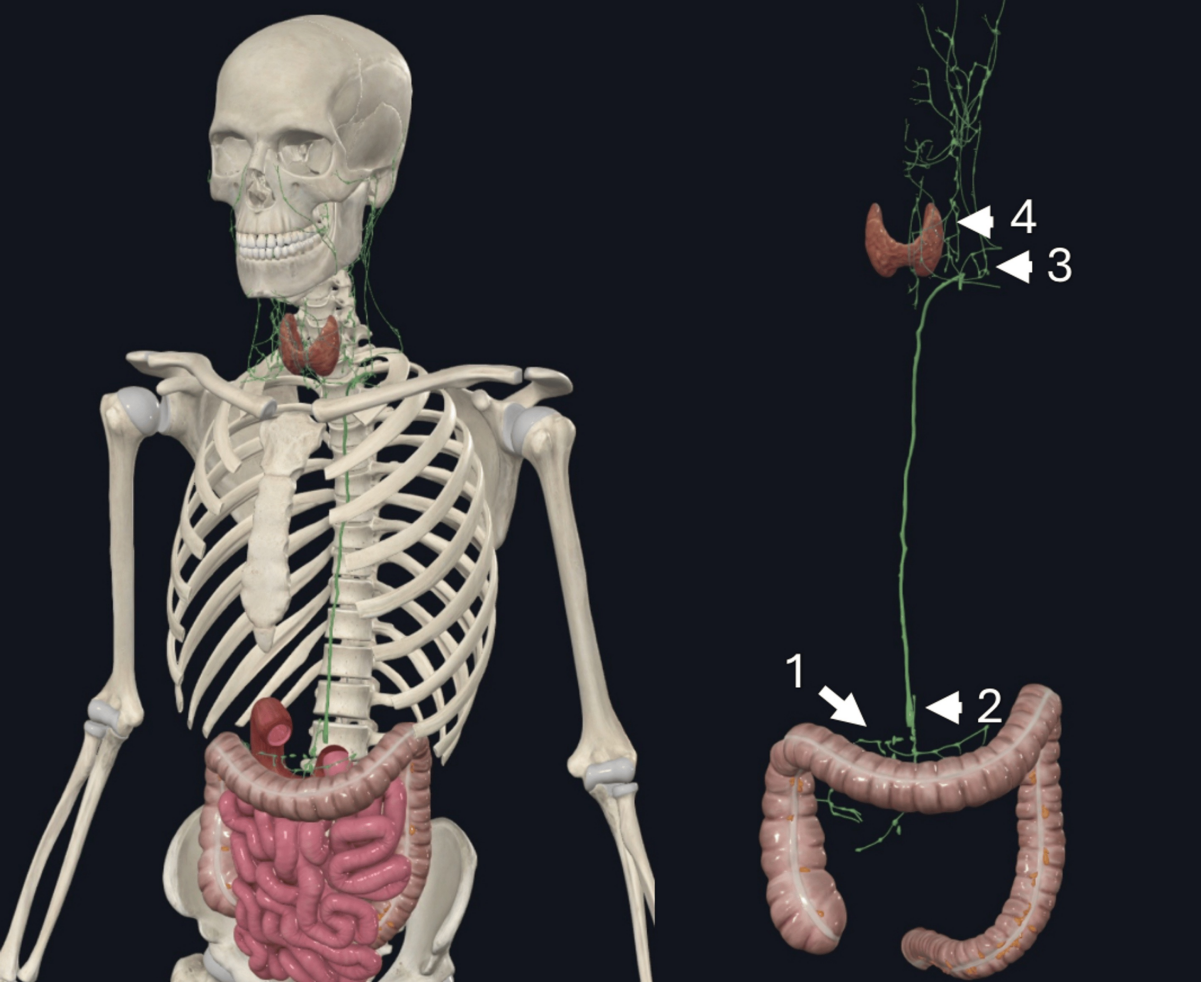
The ascending and transverse colon drain lymph into the superior mesenteric lymph vessels and nodes (1) that ultimately join the thoracic duct through cisterna chyli (2). Virchow’s node (3) is the point at which lymphatic drainage from the head and neck and infraclavicular structures join via supraclavicular lymph vessels and the thoracic duct. Obstruction here can cause retrograde metastasis to supraclavicular/head and neck structures like level IV nodes (4). Rights received to use images created by our authors using Complete Anatomy (3D4Medical Ltd, from Elsevier, Dublin, Ireland) [16]

Understanding the pathway of left supraclavicular metastasis in cases of a primary infraclavicular malignancy requires careful consideration of the role of the thoracic duct. The thoracic duct drains lymph from the lower body, abdomen, and left thorax into the venous circulation and originates from the cisterna chyli ultimately terminating at the confluence of the internal jugular and subclavian veins [14]. Retrograde flow or obstruction at this junction can spread lymph back into the left supraclavicular nodes, particularly to the most proximal left supraclavicular lymph node, located near the junction of the left subclavian vein and the thoracic duct, otherwise known as Virchow’s node [15]. Through this proposed pathway, infraclavicular malignancies, such as gastrointestinal cancers, can metastasize to the left cervical region presenting as head and neck cancer. This mechanism offers a plausible explanation for how malignancies below the diaphragm may access the lymphatic networks of the head and neck, with the thoracic duct serving as a critical route (Figure 2).

The immunohistochemical findings in this patient, positivity for CK20 and CDX2, and negativity for CK7, were crucial in confirming the diagnosis of metastatic CRC [8]. This immunoprofile is specific to gastrointestinal tissue and provided strong evidence that the left supraclavicular node was likely a metastatic lesion originating from the gastrointestinal tract rather than from an unidentified primary head and neck cancer. Incorporating immunohistochemistry into routine clinical practice, especially for patients with a history of malignancy, is vital as it guides targeted and appropriate treatment strategies based on the tumor’s origin. In this case, without these specific markers, the diagnosis might have been missed or misinterpreted, highlighting the importance of comprehensive diagnostic tools for accurately assessing unusual presentations.

Management of mucinous adenocarcinoma with metastasis requires a multidisciplinary approach, integrating surgical, medical, and radiation oncology expertise. Treatment plans must be individualized, considering the tumor’s extent, location, the patient’s overall health, and previous treatments. Imaging plays a crucial role in this process, as contrast-enhanced CT, PET-CT, or MRI scans are necessary to precisely evaluate the tumor’s spread, guide treatment decisions, and assess overall prognosis. Surgical resection remains the cornerstone for resectable metastases, offering the potential for extended survival and improved quality of life. However, in cases like this, where surgery may be complicated by the anatomical location of metastases or the possibility of other clinically undetected metastatic sites, systemic chemotherapy, and targeted therapies become critical components of management [6]. Chemotherapy regimens such as FOLFOX and FOLFIRI are commonly used in metastatic CRC to disrupt cancer cell division and DNA synthesis. The use of these chemotherapies may be supplemented with targeted therapies, such as cetuximab or bevacizumab, especially if the tumor is RAS wild-type, to enhance treatment effectiveness [9]. Accurate and surveillance imaging is essential in monitoring the tumor’s response to these treatments and in making any necessary adjustments to the therapeutic plan.

Guidelines from the American Society of Colon and Rectal Surgeons recommend surgical resection of metastasis when feasible, with chemotherapy and targeted therapies as alternatives when surgery is not an option [17].

The evaluation of MSI is another crucial aspect in guiding the management of metastatic CRC. MSI status can help predict responsiveness to immunotherapy; tumors that are MSI-H or mismatch repair deficient tend to respond well to immune checkpoint inhibitors such as pembrolizumab or nivolumab [9]. These treatments offer a more personalized approach and are critical in cases where systemic chemotherapy may not be as effective or feasible. 

The prognosis for patients with metastatic CRC remains poor, especially when metastasis occurs in rare locations such as the head and neck. The five-year survival rate for metastatic CRC is around 15%, but this figure can vary significantly depending on the sites of metastasis and the tumor’s response to treatment [2,3,5]. The limited number of cases and studies focussing on head and neck metastasis from CRC complicates the establishment of standardized treatment protocols. This case illustrates the urgent need for more extensive research to better understand metastatic pathways, such as the role of the thoracic duct, and how they might be leveraged or blocked to prevent spread. It also reinforces the importance of exploring the potential of adjuvant radiotherapy in cases where surgical resection is challenging, as it may offer additional control and symptom relief [6].

## Conclusions

This case report sheds light on the importance of considering infraclavicular malignancies such as CRC in the differential diagnosis of a left-sided head and neck mass with no identifiable primary source. Understanding these rare metastatic pathways, including potential spread through the thoracic duct, is essential for accurate and timely diagnosis, enabling prompt, evidence-based treatment. The use of immunohistochemistry in clinical practice is vital for differentiating metastatic gastrointestinal tumors from primary head and neck malignancies, guiding targeted therapeutic strategies. A multidisciplinary approach that integrates surgical, medical, and radiation oncology expertise is crucial for managing such complex cases to optimize patient outcomes.

Imaging techniques are equally important in accurately assessing the extent of the disease, monitoring treatment response, and guiding management decisions. Future research is needed to establish standardized protocols and develop tailored treatment strategies for managing rare metastatic presentations like head and neck involvement in CRC. These efforts aim to improve survival rates and quality of life, ensuring that patient care remains both effective and evidence-based.
